# Developing a Game (Inner Dragon) Within a Leading Smartphone App for Smoking Cessation: Design and Feasibility Evaluation Study

**DOI:** 10.2196/46602

**Published:** 2023-08-11

**Authors:** Justin S White, Marie K Salem, Séverine Toussaert, J Lee Westmaas, Bethany R Raiff, David Crane, Edward Warrender, Courtney Lyles, Lorien Abroms, Johannes Thrul

**Affiliations:** 1 Philip R Lee Institute for Health Policy Studies University of California San Francisco, CA United States; 2 Department of Epidemiology and Biostatistics University of California San Francisco, CA United States; 3 Department of Health Law, Policy & Management Boston University School of Public Health Boston, MA United States; 4 Department of Economics University of Oxford Oxford United Kingdom; 5 Population Science American Cancer Society Atlanta, GA United States; 6 Department of Psychology Rowan University Glassboro, NJ United States; 7 23 Limited London United Kingdom; 8 Department of Medicine University of California San Francisco, CA United States; 9 Department of Prevention and Community Health George Washington University Washington, DC United States; 10 Department of Mental Health Johns Hopkins Bloomberg School of Public Health Baltimore, MD United States; 11 Sidney Kimmel Comprehensive Cancer Center Johns Hopkins University Baltimore, MD United States; 12 Centre for Alcohol Policy Research La Trobe University Melbourne Australia

**Keywords:** smoking cessation, mobile app, games for health, gamification, software design, feasibility, mobile phone

## Abstract

**Background:**

Several stand-alone smartphone apps have used serious games to provide an engaging approach to quitting smoking. So far, the uptake of these games has been modest, and the evidence base for their efficacy in promoting smoking cessation is still evolving. The feasibility of integrating a game into a popular smoking cessation app is unclear.

**Objective:**

The aim of this paper was to describe the design and iterative development of the Inner Dragon game within Smoke Free, a smartphone app with proven efficacy, and the results of a single-arm feasibility trial as part of a broad program that seeks to assess the effectiveness of the gamified app for smoking cessation.

**Methods:**

In phase 1, the study team undertook a multistep process to design and develop the game, including web-based focus group discussions with end users (n=15). In phase 2, a single-arm study of Smoke Free users who were trying to quit (n=30) was conducted to assess the feasibility and acceptability of the integrated game and to establish the feasibility of the planned procedures for a randomized pilot trial.

**Results:**

Phase 1 led to the final design of Inner Dragon, informed by principles from psychology and behavioral economics and incorporating several game mechanics designed to increase user engagement and retention. Inner Dragon users maintain an evolving pet dragon that serves as a virtual avatar for the users’ progress in quitting. The phase-2 study established the feasibility of the study methods. The mean number of app sessions completed per user was 13.8 (SD 13.1; median 8; range 1-46), with a mean duration per session of 5.8 (median 1.1; range 0-81.1) minutes. Overall, three-fourths (18/24, 75%) of the participants entered the Inner Dragon game at least once and had a mean of 2.4 (SD 2.4) sessions of game use. The use of Inner Dragon was positively associated with the total number of app sessions (correlation 0.57). The mean satisfaction score of participants who provided ratings (11/24, 46%) was 4.2 (SD 0.6) on a 5-point scale; however, satisfaction ratings for Inner Dragon were only completed by 13% (3/24) of the participants.

**Conclusions:**

Findings supported further development and evaluation of Inner Dragon as a beneficial feature of Smoke Free. The next step of this study is to conduct a randomized pilot trial to determine whether the gamified version of the app increases user engagement over a standard version of the app.

## Introduction

### Background

Cigarette smoking is a leading preventable cause of death and disease in the United States [1]. Although many clinic-based and phone-based smoking cessation treatments are effective, most people who try to quit smoking do so without any aids and are successful <5% of the time [2]. The recommended cessation treatment, which combines counseling with medication, is used by <5% of those who attempt to quit [3].

Smartphone apps for smoking cessation have become increasingly popular among smokers who want to quit. As of 2020, English-language smoking cessation apps had been downloaded 33 million times [4]. The low cost and convenience of smartphone-delivered support makes them an appealing alternative to clinical treatments for many smokers, especially those disproportionately affected by the harms of smoking such as low-income, less educated, and racially minoritized subgroups [5]. In addition, the widespread availability of smartphones—85% of US adults owned one as of 2021—has helped extend the reach of smoking cessation apps to a large number of people [6]. Thus, smartphone-based interventions hold great promise for reducing the high burden and disparities in smoking-related cancer risk and mortality. Although many cessation apps do not follow evidence-based guidelines [7-9], a few are now being developed using established behavior change theory and existing clinical guidelines.

A perennial challenge for mobile health (mHealth) apps is their low engagement and retention rates [10]. Overall, <5% of users actively use a downloaded mHealth app daily, and <5% of users continue to use the app after 10 days [11]. Approximately half of the participants in studies of app-based mHealth interventions drop out, and studies of app-based smoking cessation interventions have found similarly high dropout rates [12-15]. Given the potential for smartphone apps to reach millions of smokers and reduce tobacco use, it is important to find ways to engage app users and increase retention and efficacy.

Behavioral researchers have suggested using serious games and gamification techniques to promote behavioral health [16-18]. Serious games are games with a primary purpose other than entertainment, such as health promotion. Gamification is a related motivation tool that uses nonmonetary rewards to make nongame activities fun or challenging. Gamification is designed to increase users’ motivation and engagement, which in turn may increase exposure and adherence to a smoking cessation program [19]. A study found that approximately 75% of the adult smokers surveyed played video games [20], and many smokers believed that game-based interventions for smoking cessation would be motivating [20,21].

Several gamified apps have been developed to help people quit smoking, such as Cigbreak [22], Crush the Crave [23,24], Inspired [25], QuitIT [26,27], Quittr [28,29], and Tobbstop [30,31]. A small number of randomized controlled trials of these apps have been conducted, and they have generally found that users were satisfied with the gamified app, but most did not experience significant increases in smoking abstinence [23,27,29,31]. User engagement and retention of these apps also varied, with each study adopting a different approach to measurement (eg, number of game “episodes” completed or minutes played). Nonetheless, nonrandomized studies have suggested that elements of gamification, such as achievement badges, may increase users’ self-efficacy and motivation or intention to quit [32,33]. Taken together, these findings demonstrate limited and conflicting information regarding the effectiveness of gamified apps for increasing smoking cessation relative to quitting without assistance, and additional efforts are needed to improve user engagement and retention.

### The Smoke Free App

Our team of academics and software developers sought to design and integrate a game into the Smoke Free smartphone app. Developed by London-based 23 Limited, Smoke Free is one of the most downloaded smoking cessation apps in the Apple and Android stores, with >800,000 downloads per year and 6 million downloads so far [34,35]. A preprint of a pragmatic randomized controlled trial reported that, although being randomized to receive an offer to use the Smoke Free app did not increase smoking abstinence compared with no intervention, the app increased continuous abstinence at 6 months when smokers who were randomized to receive the app downloaded it (12.7% intervention vs 7% comparator) [35]. The app leverages behavior change techniques that have proven to be effective in face-to-face behavioral support programs [36]. Key features of the app include (1) a calculator that tracks the total amount of money saved by not smoking; (2) a calendar that tracks the time elapsed since the user quit smoking; (3) a scoreboard that awards badges to users for not smoking; (4) progress indicators that inform users about health improvements that the user can expect because they started their quit attempt; (5) daily missions that assign evidence-based tasks to help users avoid and resist urges to smoke; (6) a diary and cravings log that track the frequency, strength, and location of cravings to smoke; and (7) a text-based chatbot that delivers quitting guidance in a friendly, conversational tone. In randomized trials, a full version of the app with chatbot was shown to increase user engagement compared with a reduced version of the app [37], and the daily missions were shown to increase user retention and self-reported smoking abstinence at 3 months [34]. The current standard version of the Smoke Free app does not contain gamification features apart from the virtual badges noted previously.

### The Inner Dragon Game

**Figure 1:**
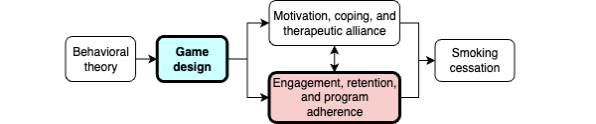
Conceptual model.

Our team designed a theory-informed game called Inner Dragon and integrated it into the Smoke Free smartphone app. Inner Dragon uses game elements that incorporate behavioral insights to increase user engagement and retention. This increased engagement is expected to increase exposure and adherence to the smoking cessation program, which should improve the chances of quitting smoking (Figure 1). The process of developing Inner Dragon and the game design is described in the *Methods* section.

This feasibility trial is a precursor to a 2-arm pilot randomized controlled trial and part of a broad program that seeks to assess the effectiveness of gamifying an app for increasing smoking cessation. The pilot trial will evaluate the engagement, satisfaction, and early efficacy of gamifying the Smoke Free app compared with a standard version of Smoke Free to guide the design of a large trial. The aim of this study was to assess the feasibility and acceptability of adding the Inner Dragon gamification component to the existing Smoke Free smartphone app for smoking cessation.

## Methods

### Overview

**Figure 2:**
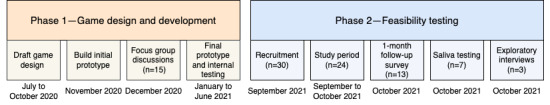
Overview of study approach.

The study was conducted in 2 phases. In the first phase, the study team designed and integrated the game into the Smoke Free app. In the second phase, the study team evaluated the feasibility and acceptability of the integrated game using a single-arm trial. An overview of the study approach is provided in Figure 2. Our approach follows the biphasic framework for gamification design by Marczewski [38], involving a first phase of planning and design based on iterative information collection and development and a second phase of measuring user activities and engagement [39].

### Phase 1—Game Design and Development

We followed a multistep usability testing procedure for the development of the Inner Dragon game: (1) we created a game design document and sketches of app screens for the game; (2) we conducted focus group discussions (FGDs) to gather feedback about sketches with Smoke Free users who are not participating in the feasibility trial; and (3) we finalized the game prototype, integrated it into Smoke Free, and performed internal app testing.

#### Game Development

As an initial step, we partnered with the game design studio, Workinman Interactive, LLC, to produce a game design document that described the significant features of the game. Workinman has a long history of developing games for apps and clients include Disney, Fisher-Price, and NBCUniversal. The investigative team provided an initial concept for the game. The game design document was refined based on a series of meetings and multiple rounds of revisions. Workinman also provided supporting art for wireframes, game flowcharts, and screen images. These materials were used by 23 Limited to build an initial prototype of the Inner Dragon game.

#### Design of Inner Design

In the Inner Dragon game, users care for a customizable pet dragon, whose growth reflects the user’s own progress in quitting smoking. The game uses traditional virtual pet retention mechanics with some social features and many options for customization and personalization. Virtual pets have been popular with consumers because they can foster bonding and companionship [40]. The Inner Dragon concept is also broadly compatible with the genres of games most liked by adult smokers, namely action, role-playing, and action-adventure games [41].

**Figure 3:**
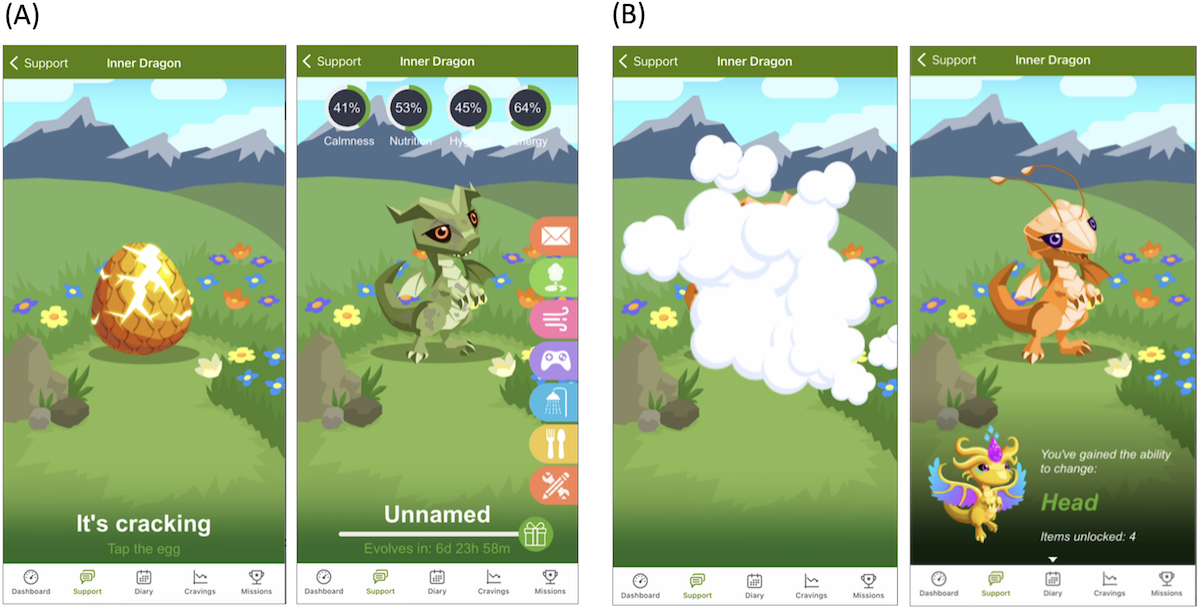
Dragon evolution. (A) Egg hatches into full dragon. (B) Evolves every 7d, gains new “abilities.”.

**Figure 4:**
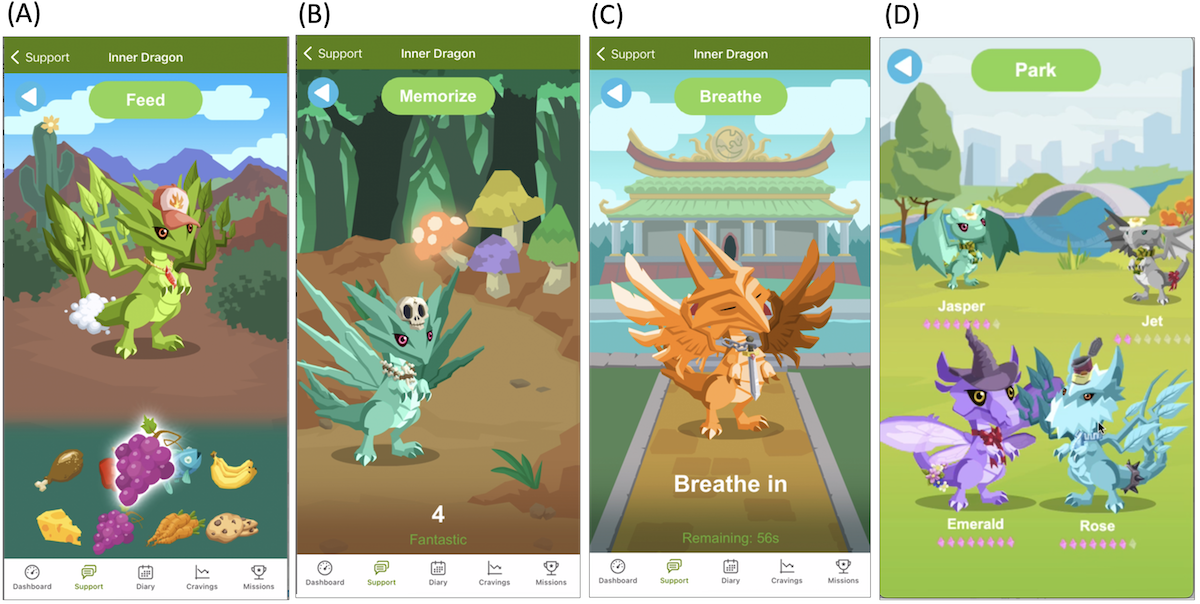
Dragon care. (A) Feeding dragon (fun). (B) Memory mini-game (distraction). (C) Breathing exercise (manage cravings). (D) Dragon park (social feedback and support).

Inner Dragon includes several game mechanics designed to increase user engagement and retention, with the primary goal of increasing exposure and adherence to the smoking cessation program, thereby improving the likelihood that the user quits smoking. Inner Dragon allows users to interact with a digital pet that is tied to their quit attempt as a means of progress toward continued abstinence, positive reinforcement for engagement with the smoking cessation program, and entertainment [40]. Game features were designed to accommodate any intensity of game play, whether heavy or sporadic, with no limitation on the amount of time or engagement. The key game mechanics were as follows. First, the user maintains a pet dragon that hatches on their quit day and evolves every 7 days to unlock new attributes and powers (Figure 3). The dragon acts as a virtual avatar that represents the user’s progress. Second, the user earns experience points by engaging in selected in-app activities, including those in the game (eg, playing a minigame or feeding the dragon) and in the core Smoke Free app (eg, completing a mission or logging a diary entry). Rewards for engaging with core app features were designed to improve adherence to the smoking cessation program. The experience points unlock gifts and cosmetics, directly rewarding frequent and consistent use of the game. The user can customize their dragon (eg, wing shape or clothing accessories) over the course of the quit experience by steadily unlocking features. Third, the Inner Dragon home screen has “care meters” that users must work to keep from falling very low: calmness, nutrition, hygiene, and energy. Engaging with the dragon in various ways increases the meters. For example, petting the dragon increases calmness, and feeding the dragon increases nutrition. Caring for and interacting with a virtual pet through the care meters can foster a bond with the pet and motivate users to return to the game regularly [40]. Fourth, the game provides tools for the user to better cope with the challenges of withdrawal: a dragon-led breathing exercise to provide calmness and a memory minigame as a distraction. Fifth, a user can asynchronously interact with other users’ dragons in a “dragon park” by (1) viewing their profile and progress and (2) sending and receiving motivational messages and emojis from a preset menu (Figure 4). These game mechanics are hypothesized to combine to increase the users’ engagement with the app and, subsequently, their chance of quitting successfully.

The game design was informed by principles from the fields of psychology and behavioral economics. As noted in the following section, aspects of the game were motivated by several theories and theoretical constructs, such as self-determination theory, theory of present bias and hyperbolic discounting, theory of salience and limited attention, and variable reinforcement under operant conditioning. The avatar provides salient, visual feedback with endogenous value (tied closely to the user’s motivation to quit) associated that may sustain and enhance motivation to quit. Self-determination theory predicts that this type of feedback is highly intrinsically motivating [42]. Furthermore, the user may identify with the digital pet as an avatar, and this may cultivate a digital therapeutic alliance with the game and app, for example, by creating a bond with the dragon and increasing the user’s confidence to succeed [43,44]. The use of frequent, salient, in-game rewards was designed to counter the behavioral economic constructs of present bias and inattention to app use. The design further included evidence-based practices from contingency management, such as the use of escalating in-game rewards for abstinence, with a reset point for lapses and sustained abstinence (by harnessing regret and loss aversion) [45]. The use of surprise gifts provided a variable reward structure designed to boost engagement and novelty. The asynchronous interactions with other users in the dragon park provide opportunities for limited social support and social comparisons that may motivate the user to exert more effort in the quit attempt [46,47].

The various game mechanics and elements were designed to appeal to users with different motivations. For example, Yee [48] identified 3 main components of player motivation: *achievement* (advancement and competition), *social* (socializing and relationship), and *immersion* (discovery, customization, and escapism). The experience point system may appeal to achievers; the interaction with other players and a sense of connection with the dragon may appeal to socializers, and the rich opportunities for dragon customization and distraction game may appeal to players seeking immersion.

#### FGD Procedures

Following the development of an initial game prototype, the investigative team conducted FGDs with existing users of the Smoke Free app to gather their feedback about the Inner Dragon game concept. The findings from the FGDs were used to refine Inner Dragon in preparation for the feasibility trial.

FGD participants were recruited using a recruitment message displayed to new Smoke Free users on an onboarding screen. The message included a hyperlink to a Qualtrics screening questionnaire. Eligibility criteria included the following: aged ≥18 years; had downloaded and opened the Smoke Free app; lived in the United States; spoke, wrote, and read English; smoked at least 1 cigarette every day; and was available during one of the scheduled FGDs.

The study team organized three 90-minute FGDs via Zoom (Zoom Video Communications). During each session, a facilitator guided the participants through several discussion topics: past smoking experience, preferences for methods of quitting, and feedback about the game prototype (Multimedia Appendix 1). For the latter topic, a facilitator provided the FGD participants with a description of the different elements of the initial prototype and accompanying screenshots. Participants received a payment of US $40 as a choice of a gift card or cash card provided through the Tremendous digital payment platform.

FGD sessions were transcribed. Overall, 2 independent coders identified key themes from the transcripts. Coders then input their findings into a series of “feedback capture grids,” which organized the likes, criticisms, questions, and ideas of users for each discussion topic. Each gamification feature was evaluated with respect to its interest, ease of use, motivational potential, and overall satisfaction.

### Phase 2—Feasibility Testing

#### Overview

This study included a single-arm, unblinded evaluation of the feasibility and acceptability of the Smoke Free app with integrated Inner Dragon game, in preparation for a planned randomized pilot trial. All participants in the feasibility study were given free access to the Smoke Free app with the integrated Inner Dragon game. Participants’ use of the app was passively monitored for 28 days, and follow-up assessments occurred after 1 month.

#### Recruitment and Participants

Study participants were recruited from the population of general users of the Smoke Free app. Focus group participants were not included. New users of Smoke Free were invited to participate in a feasibility trial to quit smoking through an on-screen recruitment message presented on the initial onboarding screens within the app. The message asked the following: “Interested in a research study to test the latest version of Smoke Free? If you qualify you could earn up to US $100 and get some paid features for free!” The message included a hyperlink to a screening questionnaire and consent form in the Qualtrics web-based survey platform. Recruitment remained open until the prespecified sample size of 30 participants was met.

Inclusion criteria were the following: aged ≥18 years; downloaded and opened the Smoke Free app; lived in the United States; spoke, wrote, and read English; smoked at least 1 cigarette every day; planned to quit smoking within the next 7 days; and was willing to self-administer a salivary cotinine test remotely. At the end of the screening questionnaire, eligible respondents were asked to consent electronically to participate in the study. Respondents’ eligibility data were automatically transmitted to Smoke Free’s servers to designate each person as included in or excluded from the study. Following the completion of the remaining onboarding process in Smoke Free, eligible, consenting users were automatically given immediate access to Inner Dragon and certain core (ie, nongame) features of the app. Core features included all of those described previously, including the chatbot and daily missions, which are typically paid features.

#### Procedures

During onboarding, each participant in the feasibility trial selected their own planned quit date within 7 days of enrollment. Participants were invited to use the Smoke Free app in whatever way they liked for 28 days after their planned quit date. Therefore, all participants had the same amount of time with the app following their initial quit date. Use was self-directed and did not require any specific amount of time.

We collected self-administered questionnaire data from participants via Qualtrics at baseline and weekly on days 7, 14, 21, and 28 after the participant’s planned quit date. After completing the screening questionnaire, meeting the inclusion criteria, and completing the informed consent in a web-based form, participants were automatically sent an email link to the baseline questionnaire. The 28-day (“1-month”) follow-up assessment was the primary end point. Participants were sent 3 SMS text messages (1 invitation and 2 reminders) and 1 email reminder for the 1-month follow-up survey, using multiple modalities to try to increase response rates.

All participants who completed the 1-month questionnaire, regardless of smoking status, were invited to complete a salivary cotinine test to biochemically verify their smoking status. Within 2 business days of completing the 1-month follow-up questionnaire, participants were mailed a salivary cotinine test kit and test instructions by 2-day priority mail. We sent an email to participants with a link to a Qualtrics survey in which they were asked to upload a photo of the test results within 7 days of receiving the test kit.

Following the end of the saliva testing period, a randomly selected subset of participants was invited to participate in an exploratory 45-minute semistructured interview via Zoom to share their experiences with using the Smoke Free app.

Participants were eligible for up to US $95 in compensation. They received US $10 for completing the baseline questionnaire within 48 hours; US $5 for completing the weekly surveys on days 7, 14, and 21 within 48 hours; US $30 for completing the 28-day survey within 48 hours of being invited (or US $20 for completing it within 7 days); and US $40 for uploading a photo of their saliva test result within 48 hours of receiving the test kit (or US $30 for uploading a photo within 7 days). All study payments were delivered via email at the end of the study through Tango Card [49], a web-based gift card system that allowed participants to choose from a menu of Visa prepaid cards and hundreds of gift cards.

#### Outcomes

The study’s outcome measures were selected principally to establish the feasibility and acceptability of the intervention and data collection methods for a planned pilot trial. The study had two primary outcome measures, both focused on user engagement: (1) the total number of unique app sessions from enrollment through 28 days after the user’s initial quit date, measured as the number of times the app was opened, and (2) the mean duration of app sessions, in minutes, through day 28 after the quit date. Both outcomes were passively collected by the app. A new session was defined as opening the app after at least 30 minutes of inactivity.

Secondary outcomes focused on the feasibility and acceptability of the gamified app in the domains of (1) user engagement and retention and (2) user satisfaction. Secondary outcomes were the number of unique days with ≥1 app session; proportion entering Inner Dragon; and satisfaction with Smoke Free (“I liked using the Smoke Free app”) and Inner Dragon (“I liked the dragon game”), reported on a 5-point Likert scale ranging from “Not at all” (score=1) to “Extremely” (score=5).

Tertiary outcomes included the following: (1) the proportion of participants who reported abstaining during the past 7 days at the 1-month follow-up assessment (self-reported 7-day point-prevalence abstinence); (2) biochemically verified 7-day point-prevalence abstinence at the 1-month follow-up assessment, obtained from uploaded results for a self-administered salivary cotinine test (Alere iScreen Oral Fluid Device); (3) program adherence, measured as the number of times of using selected core app features (reported a craving, recorded a diary entry, completed a mission, read a tip, and used the chatbot); (4) number of times of using selected game features (breathing exercise, cleaning the dragon, feeding the dragon, memory minigame, using the customization menu to change appearance, and reading the dragon instruction guide); (5) motivation to quit at the 1-month follow-up, reported on a 10-point scale ranging from not at all motivated (score=0) to very motivated (score=10); and (6) digital therapeutic alliance, measured from the bonding and confidence subscales of the Mobile Agnew Relationship Measure, reported on a 4-point measure and dichotomized at a threshold of “Agree” or higher [43,44].

#### Data Analysis

We started by calculating the descriptive statistics of demographic and smoking factors. For the primary aim, descriptive statistics were used to estimate user engagement with the app. A complete case analysis was conducted, including only those who opened the app after completing the screening questionnaire.

To explore the association between the total number of app sessions and the number of sessions with game use, we estimated an unadjusted linear regression of the relationship between the total number of app sessions and the number of sessions with game use. The total number of app sessions is equivalent to the total number of sessions with use of core features, because each session starts on the main dashboard screen that includes certain core features (eg, calculator of money saved by not smoking).

Smoking abstinence was assessed using both complete case analysis and assuming that nonrespondents were still smoking (ie, “missing = smoking”). Imputation methods were not used because of the small sample size.

As this was a feasibility study, the target sample size of 30 participants was based on budget and feasible accrual during the study timeline, rather than on a power analysis. Quantitative analyses were performed using Stata (versions 16.1 and 17.0; Stata Corporation).

#### Exploratory Qualitative Interviews

To assess the opinions about and use of the Smoke Free app with Inner Dragon, we invited the feasibility study participants to participate in a 45-minute semistructured interview via Zoom. The exploratory interviews occurred following the saliva testing period. Interview respondents received a US $50 gift card of their choice. The interview included topics about overall app experience, Inner Dragon game experience, and study and data collection processes (Multimedia Appendix 2). The qualitative interview data were compiled and summarized into key points.

### Ethics Approval

The institutional review board of the University of California, San Francisco, approved study procedures for phase 1 and phase 2 (19-29335).

## Results

### Game Design and Development

In total, 3 FGDs were conducted via Zoom with 15 participants, each with 3 to 7 participants, between December 4, 2020, and December 11, 2020. Although participants had varied reactions to the prototyped concept, overall, the reaction was positive. Of the 15 FGD participants, 10 (67%) strongly agreed that they would like to use the gamified app frequently. Even when users expressed doubt or dislike, most (12/15, 80%) reported that the game, as presented, would likely encourage them to check the Smoke Free app more frequently, thus supporting the main study hypothesis.

FGD participants gave feedback about several aspects of the game. They tended to be the most enthusiastic about the “dragon park” and the ability to interact with other users and avatars. FGD participants wanted the game to hold them accountable, including by imposing a meaningful penalty for relapse. They preferred game features that facilitated distraction from and coping with cravings over pet care features, such as feeding the dragon, that were not directly connected to the quit attempt. FGD participants wanted the game to progress alongside the dragon’s growth, for example, making leveling up more challenging as the game progresses. They tended to view the customization options as a means of distraction, rather than as motivation to continue engaging with Inner Dragon over time. Feedback from the FGDs and internal testing were incorporated into the final game design.

### Feasibility Trial

#### Sample Characteristics

**Figure 5:**
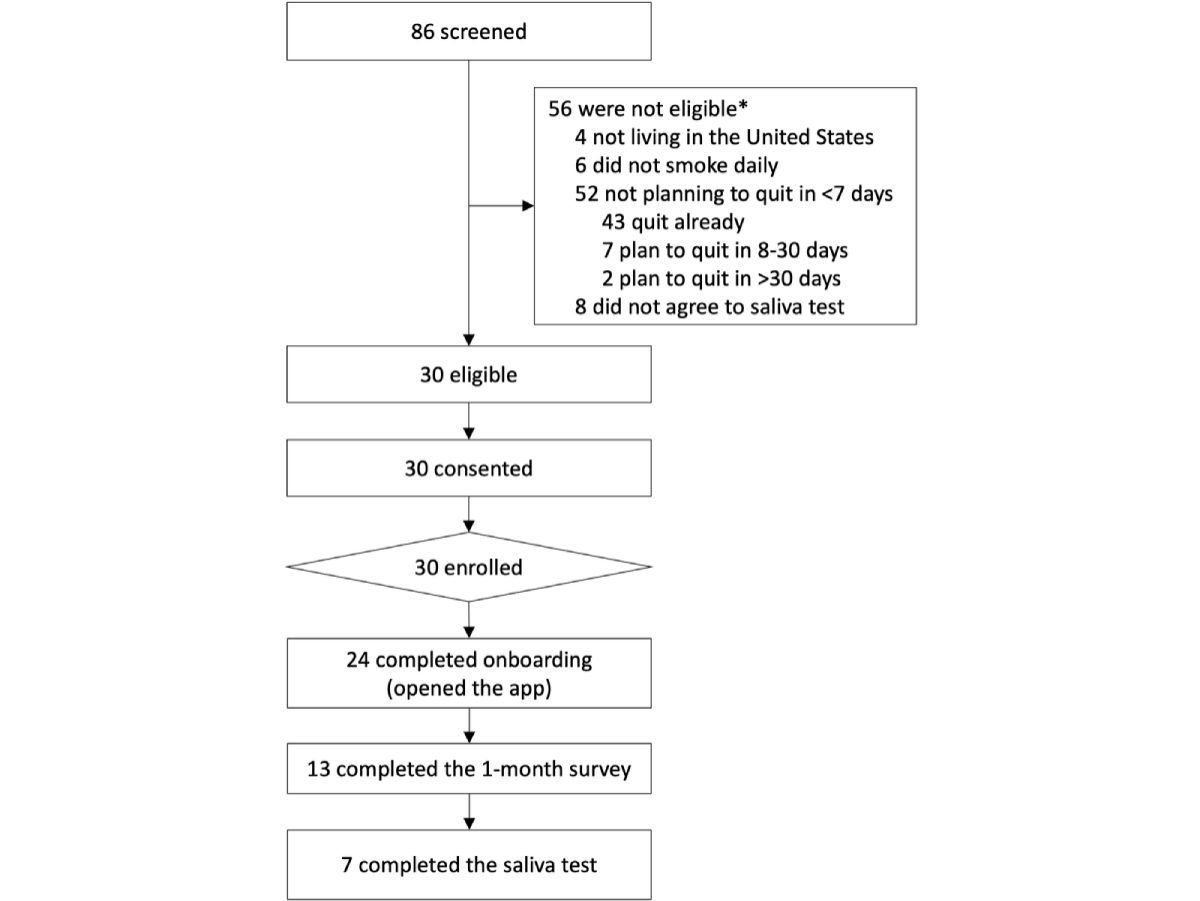
Participant flow diagram. *Individuals may be ineligible because of >1 factor.

We screened 86 new Smoke Free app users over 3 days (September 8, 2021, to September 10, 2021) to reach our goal of enrolling 30 eligible, consenting participants for the feasibility trial (Figure 5). Of the 86 screened individuals, 56 (64%) were ineligible, of whom 9% (n=5) lived outside the United States, 11% (n=6) did not smoke daily, 93% (n=52) were not planning to quit within the next 7 days (including n=43, 77% who had quit already), and 14% (n=8) did not agree to complete a saliva test. Of the 30 enrolled participants, 24 (80%) opened Smoke Free after completing the screening questionnaire, thereby completing the onboarding process within Smoke Free. This is the denominator used for most of the analyses. There was 54% (13/24) response rate for the baseline questionnaire, 46% (11/24) response rate for the 1-month follow-up questionnaire, and 45% (5/11) response rate for the saliva test (only those completing the follow-up questionnaire were asked to complete the saliva test).

Table 1 shows the demographic and smoking-related characteristics of enrolled participants. Most (17/24, 71%) identified as female, and median age was 37 years. Most (21/24, 88%) were non-Hispanic White, and 8% (2/24) were non-Hispanic Black and 4% (1/24) was Hispanic. Among the 18 baseline survey respondents, median income was US $72,500, and there was a range of educational backgrounds, with 39% (5/13) having received a bachelor’s or associate degree, 31% (4/13) having completed some college or technical school, and 15% (2/13) having received at most a high school diploma.

Participants who completed the baseline survey were asked questions about their smoking behavior (Table 1). Participants smoked a median of 15 cigarettes per day and had made a median of 5 past quit attempts. Participants had been smoking for a median of 18.5 years. Approximately 25% (3/12) of the participants reported using electronic nicotine delivery systems in the past 30 days, and 8% (1/12) reported using nicotine replacement therapy in the past 30 days. Most participants (18/24, 75%) set a quit date as within 1 day of enrollment.

Participants also reported about their past video game experience (Table 1). More than three-fourths (19/24, 79%) of the participants played video games at least monthly during the previous year, similar to the general population of smokers [20]. Moreover, 33% (8/24) of the participants played video games daily, and 33% (8/24) played at least weekly but not daily. Among participants who reported playing games monthly, mobile games were the most popular gaming platform (13/24, 54%), followed by web games (10/24, 42%).

**Table 1:** Descriptive statistics of participants^a^.

				Values
**Demographics**				
	**Gender (n=24), n (%)**			
		Female	17 (71)	
		Male	6 (25)	
		Nonbinary or transgender	1 (4)	
	Age group (years; n=24), median (IQR)		37 (26.5-43.5)	
	Household income (US $; n=13), median (IQR)		70,000 (30,000-87,000)	
	**Education (n=13), n (%)**			
		High school diploma or less	2 (15)	
		Some college or technical school	4 (31)	
		Bachelor’s or associate degree	5 (38)	
		Graduate degree	2 (15)	
	**Racial and ethnic identity (n=24), n (%)**			
		Non-Hispanic Black or African American	2 (8)	
		Non-Hispanic White	21 (88)	
		Hispanic	1 (4)	
**Smoking-related characteristics**				
	Cigarettes per day (n=13), median (IQR)		15 (10-20)	
	Past quit attempts (n=13), median (IQR)		5 (3-10)	
	Years of smoking (n=13), median (IQR)		17 (11-29)	
	Used ENDS^b^ in the past 30 days (n=12), n (%)		3 (25)	
	Used NRT^c^ in the past 30 days (n=12), n (%)		1 (8)	
	Days to quit (n=24), median (IQR)		1 (0-1.5)	
**Video gaming experience (n=24), n (%)**				
	**Frequency of playing video games in the past year**			
		Daily	8 (33)	
		At least weekly but not daily	8 (33)	
		At least monthly but not weekly	3 (13)	
		Less than once monthly	1 (4)	
		Not at all	4 (17)	
	**Platform used at least monthly in the past year**			
		Mobile games	13 (54)	
		Web games	10 (42)	
		Console games	4 (17)	
		Other games	4 (17)	

^a^Some values are missing for questions asked in the baseline questionnaire. Percentages may not add up to 100% because participants selected >1 race or ethnicity.

^b^ENDS: electronic nicotine delivery system.

^c^NRT: nicotine replacement therapy.

#### User Engagement

**Figure 6:**
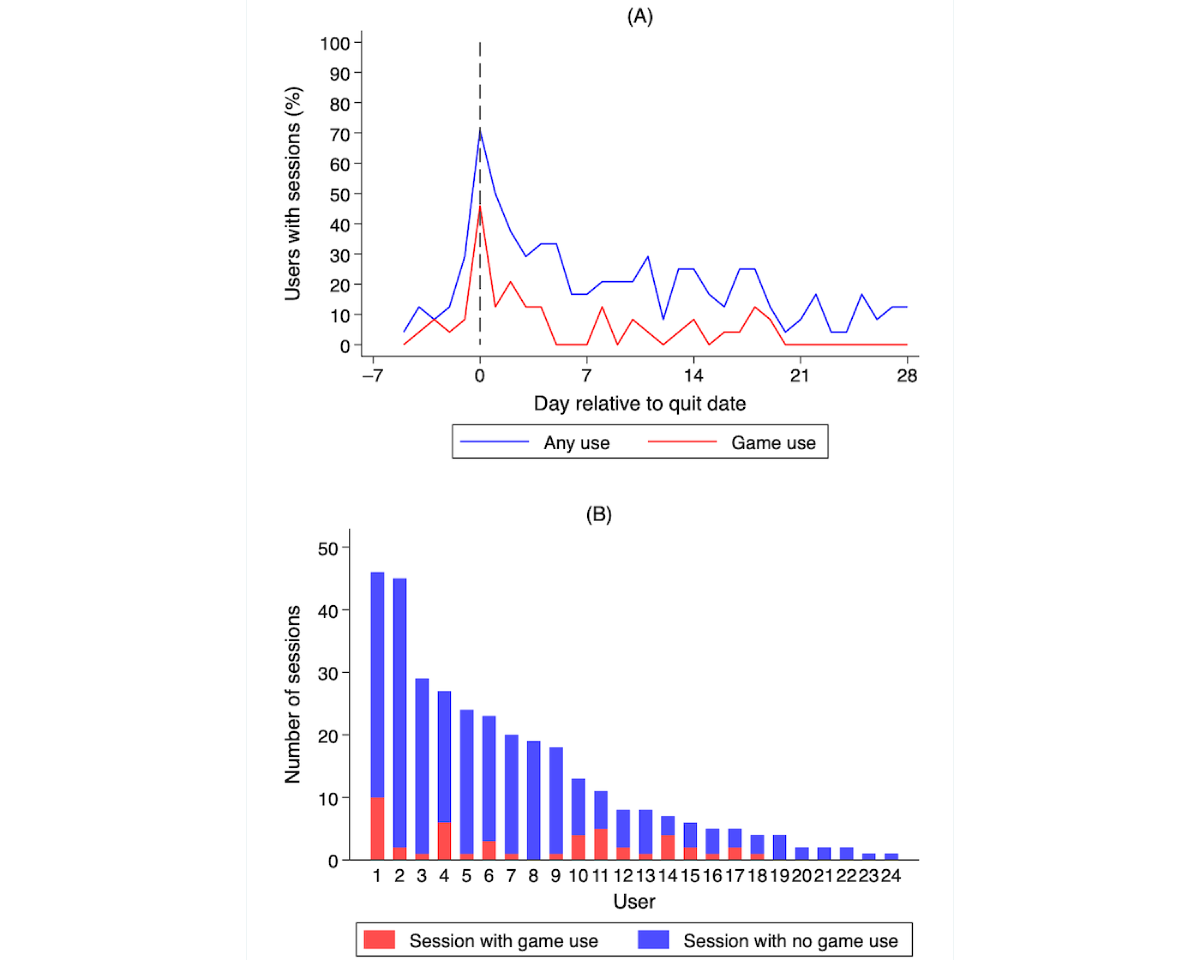
Total number of sessions. (A) Percentage of users with a session by day. (B) Number of sessions by users.

**Figure 7:**
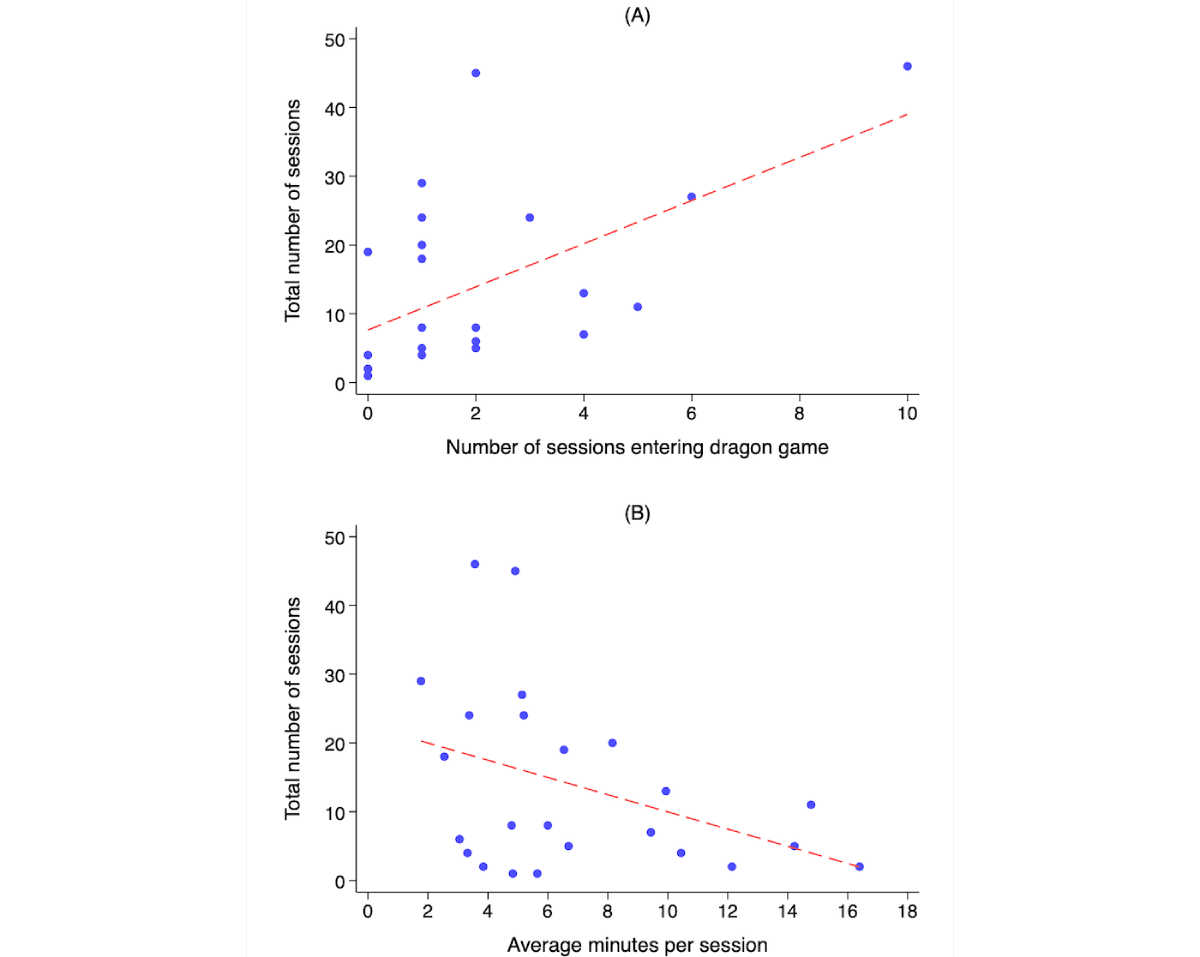
Association between number of sessions with and those without the use of Inner Dragon. (A) Number of sessions with game use. (B) Average minutes per session versus number of sessions.

User engagement with the gamified Smoke Free app was generally high: a mean of 13.8 (SD 13.1; median 8; range 1-46) app sessions per user and an average of 5.8 (SD 10.6; median 1.1; range 0-81.1) minutes per session (Table 2). Overall, 75% (18/24) of the participants entered the Inner Dragon game at least once. The number of sessions decreased rapidly during the first week after the participant’s quit date, for sessions overall and those involving the use of Inner Dragon (Figure 6A). The decline in sessions with game use was similar to the decline in sessions overall, based on the change over time in the share of sessions with game use. The high variability in the total number of sessions by user is shown in Figure 6B. The use of the Inner Dragon game was positively associated with the total number of app sessions (correlation 0.57), such that each additional session with the use of Inner Dragon was associated with 3.1 (SE 0.96) more total sessions (*P*=.003; Figure 7A). In addition, the average time a user spent per session decreased with the total number of sessions for that user (correlation −0.39), such that each additional session was associated with 1.25 (SE 0.63) fewer minutes per session on average (*P*=.06; Figure 7B).

**Figure 8:**
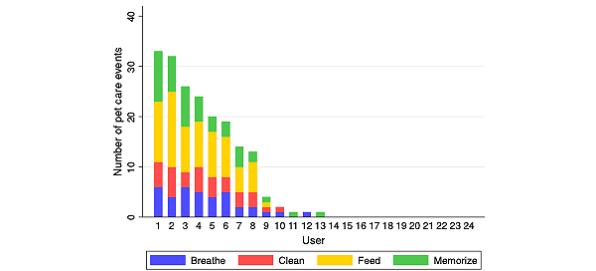
Pet care events by user.

We further characterized the variability in the use of different features of the game. Figure 8 shows the use of various pet care activities in the game. Only 44% (8/18) of the participants who tried Inner Dragon used these pet care activities with some frequency.

**Table 2:** App use during the intervention.

				Participants, n (%)		Values, mean (SD)		Values, median (range)
**App sessions (n=24)**								
	Sessions per user^a^			24 (100)		13.8 (13.1)		8 (1-46)
	Minutes per session per user^a^			24 (100)		5.8 (10.6)		1.1 (0-81.1)
	Unique days with ≥1 session^b^			24 (100)		6.8 (5.9)		4 (1-19)
	Any use of the Inner Dragon game^b^			18 (75)		—^c^		—
	Sessions with any game use per user^d^			24 (100)		2.4 (2.4)		2 (0-10)
	**Pet care events^d,e^**							
		Breathing exercise	24 (100)		1.8 (2.1)		1 (0-6)	
		Cleaning the dragon	24 (100)		1.6 (1.9)		1 (0-6)	
		Feeding the dragon	24 (100)		3.3 (4.6)		0 (0-15)	
		Memory game	24 (100)		2.1 (2.8)		1 (0-10)	
	Dragon customizations^d^			24 (100)		7.2 (15.4)		0 (0-61)
	Read the dragon guide^d^			24 (100)		4 (4.1)		1.5 (0-12)
**Efficacy (7-day abstinence at 1 month)**								
	Self-reported, complete cases (n=11)^b^			5 (45)		—		—
	Self-reported, missing cases (=smoking; n=24)^b^			5 (21)		—		—
	Verified, complete cases (n=11)^d^			3 (27)		—		—
	Verified, missing cases (=smoking; n=24)^d^			3 (13)		—		—
**Motivation and satisfaction (n=24)**								
	Satisfaction—“I liked using the Smoke Free app”^b,f^			11 (46)		4.2 (0.6)		4 (3-5)
	Satisfaction—“I liked the dragon game”^b,f^			3 (13)		2.0 (1.0)		2 (1-3)
	Motivation to quit (0-10)^d^			11 (46)		8.8 (1.7)		10 (5-10)

^a^Primary outcome.

^b^Secondary outcome.

^c^Not applicable.

^d^Tertiary outcome.

^e^Participants who did not use the pet care events, including those who did not use Inner Dragon at all, were coded as 0.

^f^Questions were rated on a 5-point Likert scale: extremely (score=5), very (score=4), somewhat (score=3), slightly (score=2), and not at all (score=1).

#### Efficacy of the Intervention

The percentage of self-reported 7-day point-prevalence abstinence at 1 month was 45% (5/11) among complete cases and 21% (5/24) when assuming that nonrespondents were still smoking. Biochemically verified 7-day point-prevalence abstinence was 27% (3/11) among complete cases who were mailed a saliva test kit and 13% (3/24) when assuming that nonrespondents were still smoking. Only 1 self-reported abstainer reported having used e-cigarettes in the past 30 days, but they did not complete the saliva test. None of the participants reported having used nicotine replacement therapy in the past 30 days.

The total number of sessions was positively associated with self-reported abstinence in a simple regression analysis. An additional session was associated with increase in abstinence of 1.3 percentage points when assuming that nonrespondents were still smoking (*P*=.04) and of 2.7% points with complete cases only (*P*=.01). However, the number of sessions with Inner Dragon use and the ratio of game sessions to total sessions were not associated with either measure of abstinence.

#### Satisfaction and Motivation

Among the 12 participants who completed the follow-up questionnaire, participants were very satisfied with the gamified Smoke Free app at the 1-month end point (mean score 4.1, SD 0.8 on a 5-point Likert scale). Satisfaction ratings were slightly higher among those who had used Inner Dragon (mean 4.2, SD 0.2; 9/12, 75%) compared with those who had not (mean 3.7, SD 0.3; 3/12, 25%); however, this difference was not statistically significant according to the Wilcoxon rank-sum test (*P*=.21). Only 25% (3/12) of the participants who had used Inner Dragon provided a satisfaction rating for Inner Dragon, because the question was not mandatory; their ratings were substantially lower (mean 2, SD 0.1) than that for Smoke Free overall.

#### Exploratory Qualitative Interviews

Qualitative interviews were conducted with 14% (3/22) of the participants who were invited and agreed to participate in the interview. Overall, 67% (2/3) of the interviewed participants had used the Inner Dragon game. In general, the participants liked the design of the app and had no major complaints. Participants found the progress indicators of health improvements as one of the most popular features, and 67% (2/3) of the participants commented about the helpfulness of the chatbot.

However, the participants reported that they had difficulty in understanding the purpose of the game and how to navigate the game. Participants also reported overlooking certain features of the game, such as the memory minigame. They recommended adding a notification to the dashboard that would link the users directly to the game and providing additional tutorials to explain the purpose of the game.

#### Adverse Events and Operational Issues

There were no adverse events reported during the trial. However, the study implementation faced technical challenges. In particular, we discovered that the dragon park had not been operational during the feasibility trial. Therefore, users were not able to use this key game feature, and user satisfaction may have been influenced accordingly. We had also planned to include a once-daily pop-up to assess daily smoking status; however, this also was not operational.

## Discussion

### Principal Findings

Drawing on multiple behavioral theories, we designed a unique, multifaceted digital pet game to promote user engagement and retention in a leading smartphone app for smoking cessation. Participants, who were given access to the gamified app, averaged 13.8 app sessions and 5.8 minutes per session throughout the 28-day trial. User satisfaction with the app was high; users reported an average score of 4.2 on a 5-point scale when asked if they “liked the Smoke Free app.” Regarding smoking abstinence, 46% (5/11) of the participants reported having abstained from smoking during the previous 7 days, and 27% (3/11) of the participants were biochemically verified to have abstained.

The development process demonstrated the feasibility of integrating the Inner Dragon game within the Smoke Free app. The single-arm trial demonstrated the feasibility of performing planned procedures for a pilot randomized controlled trial and provided proof of concept that the Inner Dragon game might be able to increase the engagement of Smoke Free users. The evaluation found that user engagement with the gamified Smoke Free app was generally high, with considerable variability across participants. Short-term data about app use suggested that users who used the game more frequently were also more likely to use core app features more frequently. A randomized controlled trial could reveal whether this is a causal relationship. Participants in the feasibility evaluation liked the gamified app overall; however, we did not collect sufficient data to gauge their satisfaction with the Inner Dragon game.

Our study makes 2 key contributions to the literature about serious games and gamification for smoking cessation. First, although several existing games such as Tobbstop and Quittr use similar frameworks and game features to promote engagement and smoking cessation, our use of a digital pet is novel for smoking cessation apps. Second, we use the game in a unique context in which it is integrated into an app with 6 million downloads so far and available at a scale that is rarely achieved by serious games.

### Comparison With Previous Studies

Our study of Inner Dragon contributes to the developing literature about the feasibility, engagement, and effectiveness of gamified smoking cessation apps. A small number of such apps have been developed and evaluated, including Crush the Crave [23,24], QuitIT [26,27], Quittr [28,29], and Tobbstop [30,31]. Overall, the literature highlights low user engagement for gamified smoking cessation apps. Crush the Crave participants reported an average rating of 2.4 out of 5 for their frequency of using the game [23]. QuitIT users also had low user engagement, with only 40% of participants reporting that they played the game [27]. User engagement was high among the Quittr users with access to the Tappy Town game (mean of 58.4 min of using the app over a 28-day trial) [29] and the Tobbstop app in which 65.9% of participants successfully used the app [31]. In comparison, 75% (18/24) of our study’s participants used the Inner Dragon game in the Smoke Free app at least once throughout the 28-day trial, resulting in relatively high user engagement compared with other apps.

A small number of randomized trials have been conducted to evaluate the efficacy of gamified apps to improve smoking cessation. Although participants have generally reported high satisfaction with the gamified app, most of the apps have not demonstrated improved smoking abstinence rates [23,27,29,31]. Our feasibility trial found a point-prevalence abstinence that exceeded those reported for most other gamified smoking cessation apps (eg, 4% for Quittr, 14% for Crush the Crave, and 39% for Tobbstop); however, the gamified Smoke Free app will need to be tested in a randomized trial with a control group.

### Future Directions

The feasibility evaluation produced several important findings with implications for the planned pilot randomized controlled trial and for future studies more generally. Several lessons relate to the game design. First, users reported overlooking the game entirely or not remembering to open it. To increase the visibility of the game, we subsequently included a message at the top of the main dashboard that linked to the game. A message linking to the game was also added under the *support* tab, where many other resources, such as the chatbot, were available. Second, to address participants’ misunderstandings regarding the purpose of the game and the availability of game features, we created and embedded a promotional video that plays when the user opens the Inner Dragon screen for the first time. The video describes the purpose of the game and its relationship with the user’s quit attempt and highlights the key features of the game, such as the breathing exercise. We also added and expanded on tutorials to clarify the availability and purpose of certain features. Third, we provided tight integration between Inner Dragon and the core features of Smoke Free through the use of more salient messaging outside the game and navigation buttons to the game. In particular, we added messages on nongame screens to inform participants when they earned experience points for completing core activities. For example, upon logging a diary entry, a message popped up to inform the user that they had won experience points that can be claimed in Inner Dragon, along with a button to navigate directly to Inner Dragon.

The feasibility trial also provided lessons for improving the study design of the planned pilot trial. The low response rates pointed toward a need to send reminder messages regarding questionnaires via SMS text message rather than via email. There was also a need to devise procedures for monitoring which app features were active.

### Study Limitations

The study has important limitations. First, the small sample size limited the precision of estimates for main outcomes. Second, and relatedly, low response rates and the potential for selection bias made it difficult to assess certain outcomes, such as user satisfaction. However, our experience allowed us to formulate new procedures to improve participant retention. In response, we have planned a large pilot trial with 500 participants to mitigate this small sample size limitation. Finally, the follow-up period was short, and a long follow-up was implemented in the next phase of the project. Several game features, such as the dragon evolutions, converting experience points into gifts, and social interactions in the dragon park, provide the basis for promoting long-term engagement with the app, and this will require further design and development.

### Conclusions

In summary, this trial showed that it is feasible to integrate a gamification component into an existing smartphone app for smoking cessation. Moreover, we developed a set of study procedures that could feasibly be used to evaluate the game intervention. Digital gamification is a promising strategy that merits further attention from smoking cessation researchers.

## Appendix

Multimedia Appendix 1 Focus group discussion guide.

Multimedia Appendix 2 Exploratory interview guide.

## Abbreviations

FGD: focus group discussion
mHealth: mobile health

## Data Availability

The survey data sets generated and analyzed during this study are not publicly available but are available from the corresponding author upon reasonable request. The app use data generated and analyzed during this study are not publicly available owing to a third-party agreement between the University of California, San Francisco, and 23 Limited (the developer of Smoke Free) that prohibits data sharing.
