# Supplementary material for: Developing a Game (Inner Dragon) Within a Leading Smartphone App for Smoking Cessation: Design and Feasibility Evaluation Study
Source: JMIR Serious Games. 2023 Aug 11;11:e46602. doi: 10.2196/46602 (PMC10457699; doi:10.2196/46602)
Supplement: Multimedia Appendix 1 [file games_v11i1e46602_app1.pdf]

Gamification App for Smoking Cessation
Focus Group Discussion Guide

[Moderator 1] = Johannes
[Moderator 2] = XYZ
[Moderator 3] = ABC
[xx] = *provides directions on how to use PPT, where applicable*

WELCOME:
[Moderator 1 - make sure to have Section 3 PPT open on your desktop]

[Moderator 1]
Hi everyone, we are @Johannes, @XYZ, and @ABC. @Johannes is a Psychologist and professor at Johns Hopkins Bloomberg School of Public health, @XYZ is XYZ, and @ABC is ABC. Thank you for agreeing to help us with our study. As you probably know, we are interested in finding out how we can best help people quit smoking. So we'd like to ask you some questions about that. The discussion will take about 90 minutes and at the end you will receive a $40 gift card, as our thank you for participating.

<u>Group Guidelines</u>

It's always a good idea for a group like ours to have some guidelines that we all agree to follow. We'll go over ours now.

1) First, your participation in this discussion today is completely voluntary. This means that you can stop being part of it at anytime. You do not have to answer a question if you don't want to. There's no penalty if you leave early, but you won't be eligible for the $40 gift card.
   - That said, a benefit of participating is that each of you has the chance to provide your opinion and information that can help our project improve the services available to someone like you who might be interested in quitting smoking.

2) There are no right or wrong answers.
   - In fact, we welcome all points of view, whether you agree or disagree with something said. Hearing about your unique experiences is the only way we'll know how well our program works for different people.
   - Please feel free to share your opinion.
   - We are interested in both positive and negative comments. Negative comments can be just as helpful as the positive comments.

3) Your privacy is very important to us
   - I want to assure you that whatever you say here will be used only for research purposes.
   - While we will be recording this discussion, your name or any identifying information will not be linked to what you say.
   - Only the research team will have access to your identifying information and it will be destroyed when the project is over.
   - Your names will not appear on any reports or presentations from this discussion.
   - Please do not share information discussed in this group with others outside of the group.

Do you have any questions about participating in this group discussion today?

Start the recording. Thank you, I'm going to start the recording now.

OUTLINE IN THE BEGINNING:

[Moderator 1]
Hi everyone, again we are @Johannes, @XYZ, and @ABC. Great to have you here – thanks for joining! Who is here with us? Just a quick hello is fine.

[Moderator 2]
Hi everyone – thanks for joining! Johannes, thanks for getting us started. Here is how today will work:

- We will ask a number of questions. We will ask the question out loud and @ABC will paste it into the Chat box (@ABC is moderating the chat for us today). You can find the Chat box by clicking on the button that says Chat at the bottom of Zoom.
- Let's make sure everyone is able to use the Chat box. Can you click on the Chat button and then type a quick hello?
- Feel free to answer either by speaking out loud or by typing into the Chat box, although we encourage you to speak out loud to make the discussion more interactive.
- The questions in the chat will be numbered, so you can follow along. We'd love to see you respond to all questions, if possible.

[Moderator 1]
Any questions about any of this?
If not, then we will go ahead and start.
Reminder that we would love to see responses to all questions if possible.

[Moderator 1]

I. PAST EXPERIENCE

For these first few questions we'd like to know about your experience with smoking and quitting.

QUESTION 1. *Smoking history.* How and why did you start smoking, and how long have you been smoking? And do you have any past experience with trying to quit smoking?We just want to hear your own story, whatever it is.

Probe for the following:

- *Why were you not able to quit in the past?*
- *What methods did you use to try to quit?*

QUESTION 2. *Quit motivation.* On a scale from 1 (not at all) to 10 (extremely), how motivated are you to quit smoking right now?

Probe for the following:

- *Why do you want to quit smoking?*
- *What could increase your motivation?*
- *What could get in the way?*

[Moderator 1]
II. **FOR PREFERENCES QUITTING**

Next, we will ask about smartphone apps as a possible way to help with quitting smoking. We will also ask questions about smartphones games in general.

QUESTION 3. *Use of smartphone apps.* What kinds of apps do you use most often on your phone and why? Any games?

QUESTION 4. *Use of Smoke Free app*. What has been your experience with the Smoke Free app so far?

Probe for the following:
- *What made you decide to use the Smoke Free app?*
- *How long have you used the app?*
- *What did you like or not like about the program?*

[Moderator 2]

III. PROTOTYPES

The Smoke Free app may add a game inside it to help users to quit smoking. Next, we will ask you some questions about the specific game that Smoke Free may add.

QUESTION 5. Next, I would like to show you examples of a new game that would be included within the Smoke Free app. I would like to hear what you think of example screens from the new game.

[Moderator 1 should now begin sharing their screen]

[Keep PPT on title page]

Here is our current idea. Smoke Free users will care for an animated pet dragon, which will grow along with you as you quit smoking. As you use the Smoke Free app and play with the dragon, you will earn points that can be used to grow the dragon and unlock accessories to customize your dragon.

a. *Interest*. Would you be interested in Smoke Free if it had a game like this? Why or why not?

Before we go on, please note that there are many aspects of the idea that are not final. We will be showing you pictures of our current idea, and your input will help us to improve upon it.

We will first discuss how users' dragon could change over time as you interact with the Smoke Free app.

Then, we will talk about how users can choose features of your dragon.

Next, we will talk about how users might take care of the dragon and unlock accessories by caring for the dragon.

Last, we will talk about how users might interact with other users in the game.

QUESTION 6. *Dragon evolution.* [flip to PPT page **2**] So, we'll start with the dragon evolution. As part of the dragon evolution, the dragon will change and grow every week of your quit attempt, for example starting as an egg, hatching on your quit day, growing horns and wings to fly, and gaining the ability to breathe fire or bubbles, etc. [flip to PPT page **3,** then page **4**, then page **5**]. It will evolve on a schedule similar to what you see below. Each evolution stage is meant to reward you for reaching a new milestone within your quit attempt.

a. *Interest*. Would you be interested in this feature? Why?

b. *Graphics*. How would you rate the look of these images?

c. *Alternative Idea 1:* How would you feel about the game starting out with a sick dragon that gets healthier over time instead of an egg?

Probe for the following:

- How would a feature like this affect your motivation to use the game and Smoke Free app?
- Do you prefer the egg or sick dragon option and why?

d. *Alternative Idea 2:* How do you feel about starting out with a dragon egg a week before your selected quit date, that hatches on your quit date and grows over time? A countdown timer would indicate time until your quit day.

Probe for the following:

- How would a feature like this affect your motivation to use the game and Smoke Free app?

e. *Motivational potential.* How would a feature like this affect your motivation to use the Smoke Free app?

QUESTION 7. *Customizations.* [flip to PPT page **6**] As part of the game, you will be able to pick your dragon's name, scale color, eye color, and head shape from one of several options. It will look something like this. [flip to PPT page **7**] In addition, you will unlock the ability to pick the look of your dragon, such as the design of its wings, and what it wears.

a. *Interest*. Would you be interested in these customization? Why?

b. *Graphics*. How would you rate the look of these images?

c. *Motivational potential.* How would a feature like this affect your motivation to use the Smoke Free app?

d. *Ordering of accessories.* The customizations could be randomized or assigned in order. Randomizing the order would allow you to look different from all of the other dragons in the dragon park. Fixing the order would make it easier to show off your progress, because other users would know how many levels you've completed. Would you prefer a random order, a fixed order, or no preference? Why or why not?

e. In which other ways would you want to be able to customize your dragon?

QUESTION 8. *Pet care and leveling up.* [flip to PPT page **8**] One of the activities in the game involves caring for the pet. This involves regularly feeding, petting, and cleaning your dragon to earn points. Meters at the top of the screen track your progress, [flip to PPT page **9**] like those shown here. Pet care helps you to unlock extra accessories for your dragon.

a. *Interest in pet care*. Would you be interested in the caring for the animated dragon? Why?

b. *Graphics*. How would you rate the look of the meters?

c. *Motivational potential of pet care.* How would a feature like the pet care affect your motivation to use the Smoke Free app?

d. *Interest in accessories***.** Caring for a pet might earn you accessories that come in one of two types, as shown here [flip to PPT page **10**]: 1) body options, such as wings or

chest scales with a new design, or 2) wearables such as hats or tail rings for your dragon. Would you be interested in the accessories? Why or why not?

e. *Motivational potential of accessories.* How would a feature like the accessories affect your motivation to use the Smoke Free app?

f. How do you feel about a penalty for relapse, for example if you lose points after a relapse or your dragon is set back to an earlier stage in the evolution?
Probe for the following:
- How would a feature like this affect your motivation to use the game and Smoke Free app?

QUESTION 9. *Dragon park.* [flip to PPT page **11**] Once a day, you can take your dragon to the dragon park for a walk. At the park, you can meet other players' dragons, send messages to other dragons at the park, and receive messages from other dragons. The park would look something like this [flip to PPT page **12**].

a. *Interest*. Would you be interested in the dragon park? Why?

b. *Graphics*. How would you rate the look of this image?

c. *Motivational potential.* How would a feature like this affect your motivation to use the Smoke Free app?

d. Would you be interested in sending messages to other dragons at the park or receiving messages from other dragons?

QUESTION 10. *Additional app features.* What other ideas for the Smoke Free app do you have?

Probe for the following:
- *What features would make it more fun to use the app?*
- *What features would be helpful to quit smoking?*

QUESTION 11. *Overall assessment.* Now, thinking about the entire game*:*

a. *Interest*. How would you rate your interest in playing this game as part of a quit attempt on a scale from 1 to 10, where 10 is most interest. Why?

b. *Favorite features.* Of all the game features we discussed, which ones interest you the most? Feel free to give your top 2 or 3 in order

c. *Fit into daily activities.* Think about how you use your phone each day. How easy or difficult would it be to use an app with this design?

Probe for the following:
- *Is this an app you would continue using for more than a few days?*

d. *Motivational potential.* How would an app like this affect your motivation to quit smoking?

QUESTION 12. *System Usability Scale*. Now, we will launch a poll. Please click through it and answer the 10 multiple choice question on how useful you think the design of this game is.

Mark a number for each question. These questions refer to the Smoke Free app, including the dragon game.

	Strongly agree				Strongly disagree
1. I think that I would like to use this app frequently	1	2	3	4	5
2. I found the app unnecessarily complex	1	2	3	4	5
3. I thought the app seemed easy to use	1	2	3	4	5
4. I think that I would need the support of a technical person to be able to use this app	1	2	3	4	5
5. I found the various functions in this app were well integrated	1	2	3	4	5
6. I thought there was too much inconsistency in this app	1	2	3	4	5
7. I would imagine that most people would learn to use this app very quickly	1	2	3	4	5
8. The app seems very cumbersome to use	1	2	3	4	5
9. I would feel very confident using the app	1	2	3	4	5
10. I would need to learn a lot of things before I could get going with this system	1	2	3	4	5

[Moderator 1]

IV. WRAPPING UP

QUESTION 13. *Length of use.* How long would you be willing to use an app for smoking cessation?

QUESTION 14. *Data tracking.* How would you feel if the app shared your data with the research team for use in our research? Why?

QUESTION 15. Is there anything else you would like us to know about your past experiences or your preferences for quitting smoking that we didn't touch on in the discussion?

QUESTION 16. Those are all the questions we have for you today. Thank you for your help and sharing your opinions with us! If there is anything else you would like to tell us, or if you have any questions, you can email at **smokefree@ucsf.edu**.

To compensate you for your time, we will send you a $40 gift card by email by the end of the next business day.

Thanks everyone. Bye.
