# Supplementary material for: Developing a Game (Inner Dragon) Within a Leading Smartphone App for Smoking Cessation: Design and Feasibility Evaluation Study
Source: JMIR Serious Games. 2023 Aug 11;11:e46602. doi: 10.2196/46602 (PMC10457699; doi:10.2196/46602)
Supplement: Multimedia Appendix 2 [file games_v11i1e46602_app2.pdf]

**Inner Dragon Semi-Structured Interview**

| Participant ID: ____________ | Date: _____________ | Interviewer:______________ |
|---|---|---|

Completed by: ○ Participant ● Interviewer

**Gamification App for Smoking Cessation**
**Beta Test Interview Guide**

## INTRODUCTIONS

*[ITALICIZED OR BRACKETED TEXT IS NOT MEANT TO BE READ ALOUD.]*

*Rules of the Interview*

Thank you for agreeing to speak. My name is [interviewer name] and I am the interviewer. Today I am going to talk about your experiences with being in this study and using the Smoke Free app. The conversation will take up to 45 minutes and you will receive $50 as our thank you for participating.

Please feel free to share your opinion. We are interested in both positive and negative comments. Negative comments can be just as helpful as the positive comments. We will not share any information from this conversation with anyone outside of the study unless it is necessary to protect you, or if required by law. Your participation is voluntary, and you have the right to withdraw from the interview at any time without penalty. We ask that you not share any private, personally identifiable information during this interview such as your name or address. We will remove it from transcripts.

*Verbal Agreement to Video Record*

Before we begin, I would like to ask your permission to video-record this interview. The recording will be used for research purposes only. As I mentioned, all information from the interview will be kept private and protected, including the video recording or any transcripts made from the video recordings. Remember to remain anonymous by not saying your name at all during the interview. So, is it ok with you if we record the interview?

1. Start recording and say the initial statement to timestamp the recording:

   "This is [insert interviewer's name]. I am with participant [insert ID]. Today is MM/DD/YYYY."

### *SMOKE FREE EXPERIENCE*

First, I would like to start with your general impressions of the Smoke Free app.

2. *Understand user experience with Smoke Free design (general impressions)*
   - ➢ How was your experience with using the Smoke Free app?
   - ➢ Was there anything about the content of the app that you liked/did not like? Why?

- Was there anything about the appearance of the app that you liked/did not like? Why?

3. *Explore interest in using the app to quit smoking (probe intent/interest to use app)*
   - Do you think that this app has helped you in your attempt to quit smoking? Why/why not?
   - Would you recommend someone else wanting to quit to use this app? Why or why not?
4. *Inquire about utility of specific app features (feedback to specific features)*
   - Which features of the Smoke Free app do you think are most useful for quitting smoking?
5. *Explore barriers to app use based on their experience (app mechanics)*
   - What kind of things made it difficult to use the app?
   - Did you find anything frustrating about using the app?
6. *Prompt suggestions to make the app more useful or engaging (ideas to improve)*
   - How would you change the app to make it more fun and motivating?
   - How would you change the app to make it more helpful to quit smoking?
   - What would make you want to use it more?

***EXPERIENCE WITH INNER DRAGON GAME***

Next, I am going to ask you some questions about the dragon game in the Smoke Free app.

7. *Understand user experience and navigation with Inner Dragon (general impressions)*
   - How was your experience with using the dragon game?
   - What was your experience finding the content in the dragon game? (e.g., the font size, colors, or navigating to the dragon and through the different screens)
   - Was there anything about the content of the dragon game that you liked/did not like? Why?
   - Was there anything about the appearance of the dragon game that you liked/did not like? Why?

8. *Explore interest in using the Inner Dragon game to quit smoking (probe intent/interest to use Inner Dragon)*
   - ➢ Do you think the dragon game helped you in the process of quitting smoking? Why or why not?
   - ➢ Would you recommend someone else wanting to quit to use the dragon game? Why or why not?
9. *Inquire about utility of specific Inner Dragon aspects*
   - ➢ Which features of the dragon game did you see? What are your thoughts on each? [Probe park, breathing, memory game, cleaning, feeding, appearance customization]
   - ➢ What was your favorite feature? Least favorite?
   - ➢ What were your thoughts on the ability to change the dragon's name and appearance?
   - ➢ What were your thoughts on the points feature of the dragon game? What would you like to get more or less rewarded for?
   - ➢ Which components of the dragon game do you think were the most useful in quitting smoking?
     [Probe park, breathing, memory game, cleaning, feeding, appearance customization]
   - ➢ Are there any additional features you would like to see in the dragon game?
10. *Explore barriers to Inner Dragon use based on their experience*
    - ➢ What kind of things made it difficult to use the dragon game?
    - ➢ Did you find anything frustrating about using the dragon game?
    - ➢ Did the progression of the dragon life make sense with the collection of points? Is there anything confusing about this process?
11. *Explore situations in which using Inner Dragon was most pleasant or unpleasant (emotional impact)*
    - ➢ Was there anything that you enjoyed while using the dragon game?
    - ➢ Was there anything that you disliked while using the dragon game?
12. *Prompt suggestions to make Inner Dragon more useful or engaging (ideas to improve)*
    - ➢ How would you change the dragon game to make it more fun and motivating?

- How would you change the dragon game to make it more helpful to quit smoking?
- What would make you want to use it more?

## *COMBINATION OF APP AND INNER DRAGON*

13. *Understand user experience with combination of app and Inner Dragon*
    - How did you like having the dragon game inside of the Smoke Free app?
    - What about the combination worked well? What did not work well?

## *STUDY PROCESS*

Finally, I have a few questions about the study itself.

14. Can you talk about why you did or did not complete most of the study surveys? [Probe on baseline, weekly, endline and whether the surveys were a burden]
15. How did you find the reminder messages to take the study surveys? [Probe on whether the messages seemed overwhelming or spammy]
16. What could the study do better to encourage survey participation in the future? [Probe on time of day, # of notifications and reminders, length of surveys]
17. Was the saliva test easy to understand, use, and send back?

## *CONCLUSION*

18. Is there anything else you would like us to know about Smoke Free or the study that we have not discussed?

Thank you for your help and sharing your opinions with us! If there is anything else you would like to tell us, or if you have any questions, you can email at **smokefree@ucsf.edu**.

To compensate you for your time, you will get $50. Within the next week, you will get an email with instructions on how to claim your payment as a cash card or gift card, whichever you prefer. Thanks again. Bye.
